# Trends, variation, and clinical characteristics of recipients of antiviral drugs and neutralising monoclonal antibodies for covid-19 in community settings: retrospective, descriptive cohort study of 23.4 million people in OpenSAFELY

**DOI:** 10.1136/bmjmed-2022-000276

**Published:** 2023-01-13

**Authors:** Amelia C A Green, Helen J Curtis, Rose Higgins, Linda Nab, Viyaasan Mahalingasivam, Rebecca M Smith, Amir Mehrkar, Peter Inglesby, Henry Drysdale, Nicholas J DeVito, Richard Croker, Christopher T Rentsch, Krishnan Bhaskaran, John Tazare, Bang Zheng, Colm D Andrews, Sebastian C J Bacon, Simon Davy, Iain Dillingham, David Evans, Louis Fisher, George Hickman, Lisa E M Hopcroft, William J Hulme, Jon Massey, Orla MacDonald, Jessica Morley, Caroline E Morton, Robin Y Park, Alex J Walker, Tom Ward, Milan Wiedemann, Christopher Bates, Jonathan Cockburn, John Parry, Frank Hester, Sam Harper, Ian J Douglas, Stephen J W Evans, Ben Goldacre, Laurie A Tomlinson, Brian MacKenna

**Affiliations:** 1 Bennett Institute for Applied Data Science, Nuffield Department of Primary Care Health Sciences, University of Oxford, Oxford, UK; 2 London School of Hygiene and Tropical Medicine, London, UK; 3 Oxford Health NHS Foundation Trust, Oxford, UK; 4 TPP, Leeds, UK

**Keywords:** COVID-19, public health, community health services, therapeutics

## Abstract

**Objective:**

To ascertain patient eligibility status and describe coverage of antiviral drugs and neutralising monoclonal antibodies (nMAB) as treatment for covid-19 in community settings in England.

**Design:**

Retrospective, descriptive cohort study, approved by NHS England.

**Setting:**

Routine clinical data from 23.4 million people linked to data on covid-19 infection and treatment, within the OpenSAFELY-TPP database.

**Participants:**

Outpatients with covid-19 at high risk of severe outcomes.

**Interventions:**

Nirmatrelvir/ritonavir (paxlovid), sotrovimab, molnupiravir, casirivimab/imdevimab, or remdesivir, used in the community by covid-19 medicine delivery units.

**Results:**

93 870 outpatients with covid-19 were identified between 11 December 2021 and 28 April 2022 to be at high risk of severe outcomes and therefore potentially eligible for antiviral or nMAB treatment (or both). Of these patients, 19 040 (20%) received treatment (sotrovimab, 9660 (51%); molnupiravir, 4620 (24%); paxlovid, 4680 (25%); casirivimab/imdevimab, 50 (<1%); and remdesivir, 30 (<1%)). The proportion of patients treated increased from 9% (190/2220) in the first week of treatment availability to 29% (460/1600) in the latest week. The proportion treated varied by high risk group, being lowest in those with liver disease (16%; 95% confidence interval 15% to 17%); by treatment type, with sotrovimab favoured over molnupiravir and paxlovid in all but three high risk groups (Down's syndrome (35%; 30% to 39%), rare neurological conditions (45%; 43% to 47%), and immune deficiencies (48%; 47% to 50%)); by age, ranging from ≥80 years (13%; 12% to 14%) to 50-59 years (23%; 22% to 23%); by ethnic group, ranging from black (11%; 10% to 12%) to white (21%; 21% to 21%); by NHS region, ranging from 13% (12% to 14%) in Yorkshire and the Humber to 25% (24% to 25%) in the East of England); and by deprivation level, ranging from 15% (14% to 15%) in the most deprived areas to 23% (23% to 24%) in the least deprived areas. Groups that also had lower coverage included unvaccinated patients (7%; 6% to 9%), those with dementia (6%; 5% to 7%), and care home residents (6%; 6% to 7%).

**Conclusions:**

Using the OpenSAFELY platform, we were able to identify patients with covid-19 at high risk of severe outcomes who were potentially eligible to receive treatment and assess the coverage of these new treatments among these patients. In the context of a rapid deployment of a new service, the NHS analytical code used to determine eligibility could have been over-inclusive and some of the eligibility criteria not fully captured in healthcare data. However targeted activity might be needed to resolve apparent lower treatment coverage observed among certain groups, in particular (at present): different NHS regions, ethnic groups, people aged ≥80 years, those living in socioeconomically deprived areas, and care home residents.

**WHAT IS ALREADY KNOWN ABOUT THIS TOPIC**

- Since the emergence of covid-19, several approaches to treatment have been tried and evaluated
- These approaches have mainly consisted of treatments such as dexamethasone, which were used in UK hospitals, from early on in the pandemic to prevent progression to severe disease
- Until recently (December 2021), no treatments have been widely used in community settings across England

**WHAT THIS STUDY ADDS**

- After the roll-out of antiviral drugs and neutralising monoclonal antibodies as treatment for patients with covid-19, patients who were potentially eligible to receive such treatments were identified and the coverage of these new treatments was assessed among these patients, in as close to real time as the available data flows would support
- While the proportion of the potentially eligible patients receiving treatment increased over time, rising from 9% (190/2220) in the first week of the roll-out to 29% (460/1600) in the last week of April 2022, coverage varied between key clinical, geographical, and population subgroups

**HOW THIS STUDY MIGHT AFFECT RESEARCH, PRACTICE, OR POLICY**

- Targeted activity might be needed to resolve lower treatment rates observed among certain geographical areas and key groups including ethnic group, people living in areas of higher deprivation, and care home residents

## Introduction

Since the emergence of covid-19, several approaches to treatment have been tried and evaluated.^1^ In UK hospitals, treatments such as dexamethasone were used from early in the pandemic to prevent progression to severe disease.^2^ However, no treatments were widely used in the community, where care was largely supportive and focused on detection of need for hospital admission.^3^ In April 2021, the UK government established a therapeutics and antivirals taskforce with the aim of identifying and deploying new medicines to treat covid-19 in community settings to reduce the risk of hospital admission.^4^

On 16 December 2021, new covid-19 medicine delivery units (CMDUs) were launched across England, offering antiviral drugs and neutralising monoclonal antibodies (nMABs) as treatment to patients with covid-19 at high risk of severe outcomes in outpatient clinics or in their own homes.^5^ By December 2021, the UK government had ordered 2.25 million courses of molnupiravir, 2.75 million courses of nirmatrelvir/ritonavir (paxlovid),^6^ and 100 000 doses of sotrovimab.^7^ Initially, sotrovimab, casirivimab/imdevimab, and molnupiravir were available at these units with nirmatrelvir/ritonavir and remdesivir becoming available in February 2022. The UK government established an expert clinical group to develop criteria to support identification of high risk groups eligible for these treatments using NHS data.^8^ The NHS in England issued detailed clinical commissioning policies,^9–13^ and people identified as high risk were informed by letter that if they ever tested positive for SARS-CoV-2, they would be eligible for these treatments.

OpenSAFELY is a secure analytics platform for electronic patient records built by our group on behalf of NHS England to deliver urgent academic and operational research during the pandemic.^14 15^ Analyses can currently run across all patients’ full pseudonymised primary care records, with patient level linkage to various sources of secondary care data. Data on patients receiving antiviral drugs and nMABs from CMDUs were similarly linked and are now updated weekly with about a week’s lag time. Code and analysis is shared openly for inspection and reuse.

This study set out to identify patients registered with OpenSAFELY-TPP practices who were potentially eligible to receive antiviral drugs or nMABs in a community setting and assess the coverage of these new treatments among these patients, in as close to real time as the available data flows would support. We also describe how coverage varied between key clinical, regional, and population subgroups, and whether any treatments given could have been inconsistent with guidance.

## Methods

### Study design

We conducted a retrospective cohort study beginning 11 December 2021 (the earliest date when a patient could have tested positive to be eligible for receiving treatment when they became available from CMDUs from 16 December 2021) and ending 28 April 2022. Regular treatment coverage reports have also been produced and updated regularly with extended follow-up time using near real time data as the treatment programme progresses.^16^

### Data sources

This analysis was conducted using the OpenSAFELY-TPP platform, which executes code across records for all patients currently registered with general practices using TPP SystmOne electronic health records (EHR) software: this covers about 23.4 million people, or 40% of the English population. It includes pseudonymised data such as coded diagnoses, drug treatments, and physiological parameters. No free text data are included. These primary care data are linked, via hashed NHS numbers, to emergency department attendance and inpatient hospital spell records via NHS Digital’s Hospital Episode Statistics; national coronavirus testing records via the Second Generation Surveillance System; and the covid-19 therapeutics dataset, a patient level dataset on antiviral and nMAB treatments, newly sourced from NHSi England, derived from Blueteq software that CMDUs use to notify NHS England of covid-19 treatments. Vaccination status is available in the general practice records directly via the National Immunisation Management System (NIMS).

### Study population

We included all individuals aged 12 years or over with a positive SARS-CoV-2 test on or after 11 December 2021 and all patients with a treatment record on or after 16 December 2021, who were registered with a general practice at the time of their test or treatment. Treatment was offered from 16 December for patients with covid-19 at high risk of severe outcomes within five days of their positive test, which meant that patients who tested positive on 11 December were eligible for treatment.

### Eligibility for treatment

We identified the population who were potentially eligible for treatment as those meeting the eligibility criteria for covid-19 antiviral or nMAB treatment in the community: being a member of a high risk group (described below under high risk groups) and with confirmed SARS-CoV-2 infection (table 1). To ensure that patients were not in hospital and therefore ineligible to receive treatment, we excluded patients if they were admitted to the hospital before or on the date of their positive test and still in hospital after that date. The official criteria had two main differences from our implementation. Firstly, before 10 February 2022, infection should have been confirmed by a polymerase chain reaction test (this requirement was then relaxed to include lateral flow tests). We were not able to distinguish between lateral flow and PCR tests in all test records, and therefore included all positive SARS-CoV-2 test results across the whole study period. Secondly, having symptomatic covid-19 was part of the eligibility criteria: however, owing to difficulties in determining symptom status (ie, it was only possible to determine whether a patient’s positive test had a symptomatic flag at the time of the test, but not whether symptoms developed later), we did not implement this requirement in our analysis. We do, however, look at this in a separate sensitivity analysis, where we restricted the potentially eligible population to only those individuals with a symptomatic flag associated with their positive SARS-CoV-2 test to determine its use as an indicator of being potentially asymptomatic.

**Table 1:** Eligibility and exclusion criteria, according to the Interim Clinical Commissioning Policy (published on 24 February 2022),^12^ for outpatients with covid-19 and their use in the present study

Criteria	Criteria applied in present study*
**Eligibility criteria**	
SARS-CoV-2 infection was confirmed by PCR testing or by lateral flow test (registered via gov.uk or NHS 119)	Positive PCR or positive lateral flow SARS-CoV-2 test in the Second Generation Surveillance System (according to NHS Digital’s tests rule set logic,^8^ patients with a previous positive SARS-CoV-2 within the past 30 days were excluded; in addition, all patients admitted to the hospital 30 days before a positive test with a covid-19 diagnosis were excluded)
With covid-19 symptoms and showing no signs of clinical recovery	Not possible†
Patient was a member of a high risk group	See box 1 and supplementary material table S1, definitions of high risk groups and group names are based on published definitions and names by NHS Digital on 24 December 2021,^10^ unless otherwise noted
**Exclusion criteria**	
Requirement for hospital admission for covid-19	Not possible (people in hospital on the day of their positive test were excluded)
New supplemental oxygen requirement specifically for the management of covid-19 symptoms	Not possible
Known hypersensitivity reaction to the active substances or to any of excipients of the drug treatments (described below) as listed in their respective summary of product characteristics	Not possible

Earlier versions of the policy had some minor differences but this version was applied to the whole study period.

*Patients who received treatment (described below) were also included in the population even if they were not identified as meeting these criteria.

†It was only possible to identify whether the patient’s positive test had a symptomatic flag, but not possible to identify whether symptoms developed later; therefore, the symptomatic flag was used in a sensitivity analysis rather than within inclusion criteria.

PCR, polymerase chain reaction.

10.1136/bmjmed-2022-000276.supp1 Supplementary data

Because we were unable to implement all of the eligibility criteria, patients who received treatment (see below) were also included in the population even if they were not identified as meeting eligibility criteria (eg, having no positive SARS-CoV-2 test). All patients with records of more than one treatment in the community within two weeks of one another (potentially due to a data quality error early in the roll-out) or with an implausible treatment date (such as dates far into the future) were excluded, because we were not able to accurately determine which treatment they received and when. The number of patients included or excluded for these reasons are reported.

Box 1 **Patient groups considered at higher risk from covid-19 and to be prioritised for treatment with antiviral drugs and neutralising monoclonal antibodies, as determined by an independent advisory group commissioned by the UK Department of Health and Social Care^10^**

- Patients with Down’s syndrome
- Patients with a solid cancer, such as active metastatic cancer, or active solid cancers at any stage
- Patients with a haematological disease and stem cell transplant recipients, such as those with sickle cell disease
- Patients with renal disease, such as those with chronic kidney stage 4 or 5
- Patients with liver disease, such as those on immune suppressive treatment
- Patients with immune mediated inflammatory disorders, such as those treated with rituximab or other B cell depleting therapy in the past 12 months
- Patients with immune deficiencies* such as severe combined immunodeficiency
- Patients with HIV/AIDS with high levels of immune suppression
- Recipients of solid organ transplants
- Patients with rare neurological conditions (multiple sclerosis motor neuron disease, myasthenia gravis, or Huntington’s disease)

For further details on these criteria and how they were applied in the present study see online supplemental table S1.*This group was labelled as "primary immune deficiency" in the publication on which this analysis is based,^10^ but in the present study it is labelled according to the current NHS Digital policy (version 6).^13^

### High risk groups

The detailed recommendations on the 10 high risk groups derived by an expert clinical group (box 1) were transformed by NHS Digital into detailed logic and analytical code, including a range of codelists, which could be used on datasets they held to identify all patients eligible for treatment by CMDUs.^8^ However, NHS Digital recognised potential limitations in identifying all eligible patients using centrally held data, and freedom was permitted for CMDUs to use non-digital solutions to identify additional eligible patients locally based on the criteria outlined by the expert group. For example, patients with stage 4 chronic kidney disease would be eligible for treatment, but if the stage of disease is not coded in their primary care record^17^ they would not be automatically identified by NHS Digital; however, renal units might notify their patients with stage 4 chronic kidney disease of their eligibility for treatment. In addition, NHS Digital holds only the General Practice Extraction Service dataset, a substantially smaller derived subset of the full dataset of general practices' EHR accessible through OpenSAFELY.

Where possible, we implemented the NHS Digital logic and associated codelists in the OpenSAFELY platform to identify patients in high risk groups. Codelists were used as published with the exception of minor adaptations made to code type where codes did not exist or were erroneous in the published codelist, and to code formatting for implementation in OpenSAFELY. Further details, including all limitations in implementing any of the logic or with the codelists are detailed in online supplemental table S1. If patients had records indicating that they fell into multiple high risk groups, all groups to which they belonged were used. The covid-19 therapeutics dataset also included the high risk groups recorded by the clinician on the submitted form; if different or additional high risk groups were recorded here, patients were also assigned to these groups. At the time of this analysis, the clinician assigned high risk group for patients receiving paxlovid or remdesivir was not available.

### Treated patients

We identified the subset of patients who received treatment in the covid-19 therapeutics dataset, along with the treatment and the date they were given, restricted to those labelled as outpatients and based on treatment forms submitted as of 7 September 2022. We included first line treatments paxlovid and sotrovimab according to the most recent national policy at the time of this analysis,^18^ as well as second and third line options remdesivir and molnupiravir, respectively. Sotrovimab and molnupiravir were available from the start of the study while paxlovid and remdesivir were only available from 10 February 2022. Previous versions of the policy also included casirivimab/imdevimab, so patients who received this treatment were also included.^9^

### Key population and clinical characteristics of treated patients

We classified patients by age group, sex, NHS region of their general practice and other key personal characteristics including ethnic groups, the level of deprivation, and rurality. Ethnic group was ascertained using 270 clinical codes grouped into broad categories; White, black or black British, Asian or Asian British, mixed, other, and unknown. Deprivation was measured by the index of multiple deprivation, separated by quintiles, derived from the patient’s postcode at lower super output area level (geographical areas with an average population of 1500 people or 650 households)^19^ for a high degree of precision. Rurality was distinguished using the Rural Urban Classification^20^ and grouped into four broad categories; urban conurbation, urban city and town, rural town and fringe, and rural village and dispersed. Patients with missing information on sex, ethnic group, deprivation, rurality, or region were included as unknown. Treated patients were also described according to whether they were in other pragmatically selected groups of interest who are sometimes subject to variation in care,^21^ including autism, dementia, learning disability, serious mental illness, care home residents, housebound, clinically extremely vulnerable, and sickle cell disease. Patients were also classified by their covid-19 vaccination status (unvaccinated, unvaccinated with a record of refusing vaccination, one vaccination, two vaccinations, or three or more vaccinations).

### Consistency with guidance

For patients who received treatment but who were not otherwise identified as potentially being eligible for treatment, we reported which eligibility or exclusion criteria were not met according to the data available (ie, no positive SARS-CoV-2 test result, or not identified as a member of a high risk group). Where possible within available data, we also reported other potential inconsistencies with guidance for patients who received treatment, such as where the high risk group identified within their records did not match the high risk group associated with their treatment.

We also assessed consistency with treatment specific criteria (as detailed in online supplemental table S2), such as patients having a recorded contraindication to the specific treatment given (eg, adolescents treated with sotrovimab or remdesivir with a bodyweight of under 40 kg, online supplemental table S2), or patients treated outside the prescribed timescale (5-7 days from symptom onset, depending on the treatment; online supplemental table S2). Symptom onset date was not available, so we used positive SARS-CoV-2 tests as a proxy to estimate the extent to which patients might have been treated outside the guidance time window.

### Covid-19 medicine delivery units

Details of all CMDUs can be found on the national website.^22^ CMDU identifiers were not available in OpenSAFELY, so we used an alternative geographical grouping known as a sustainability and transformation plan. This grouping had almost a 1:1 mapping at the time of the analysis, and we used it as a proxy to identify any variation in the proportion of patients treated between CMDUs. Patients were grouped in these plans and the proportion of patients treated in each plan was calculated. Nine decile values of coverage per plan were subsequently calculated. The subset of the population covered by TPP in each sustainability and transformation plan might not be representative of the whole plan, and plans were only included if they had >10% population coverage in TPP practices. Mappings of TPP practices and sustainability and transformation plans, used to calculate the coverage, were calculated as of March 2020, and some borders and population sizes could have changed since then.

### Descriptive statistics

We generated charts showing the cumulative number of potentially eligible and treated patients per week, stratified by high risk group, and also stratified by treatment type for treated patients. We used simple descriptive statistics to summarise the numbers and proportions of potentially eligible patients treated, stratified by treatment type and either high risk group or clinical and population groups, and to describe potential inconsistencies with guidelines.

### Codelists and implementation

Information on all covariates were obtained from primary care, secondary care, and other records by searching TPP records and linked datasets for specific coded data. Detailed information on compilation and sources for every individual codelist is available at https://www.opencodelists.org/ and all codelists are available under open licences for review and reuse by the broader research community.

### Software and reproducibility

All data were linked, stored, and analysed securely through OpenSAFELY, a data analytics platform created by our team on behalf of NHS England to answer urgent covid-19 research questions (https://opensafely.org). All activity on the platform is publicly logged and all analytical code and supporting clinical codelists are automatically published at the time of results publication or sooner. In addition, the framework provides assurance that the analysis is reproducible and reusable. Further details on our information governance can be found on page 20, under information governance and ethics.

We conducted data management and analysis using the OpenSAFELY software libraries, Python 3, and R version 4.0.2. All code for the OpenSAFELY platform is freely available under open licenses for review and reuse on GitHub (https://github.com/opensafely). All code for data management and analysis for this paper is freely available under open licenses for review and reuse on GitHub (https://github.com/opensafely/antibody-and-antiviral-deployment).

### Reporting

This study followed STROBE-RECORD reporting guidelines. Charts and results not presented in this paper are available online for inspection in the associated GitHub repository.^23^ To mitigate the risk of disclosure, patient numbers of 0-7 were shown as <8 with remaining numbers rounded to the nearest 10 to protect against small number differences when routinely updating data. All percentages were calculated with 95% confidence intervals.

### Patient and public involvement

Patients and the public were not formally involved in developing this specific study design, because it was developed in the context of the rapid roll-out of a new treatment service during a global health emergency. We have developed a publicly available website https://opensafely.org/ through which we invite patients or members of the public to contact us regarding this study or the broader OpenSAFELY project.

## Results

### Eligibility for treatment

**Figure 1:**
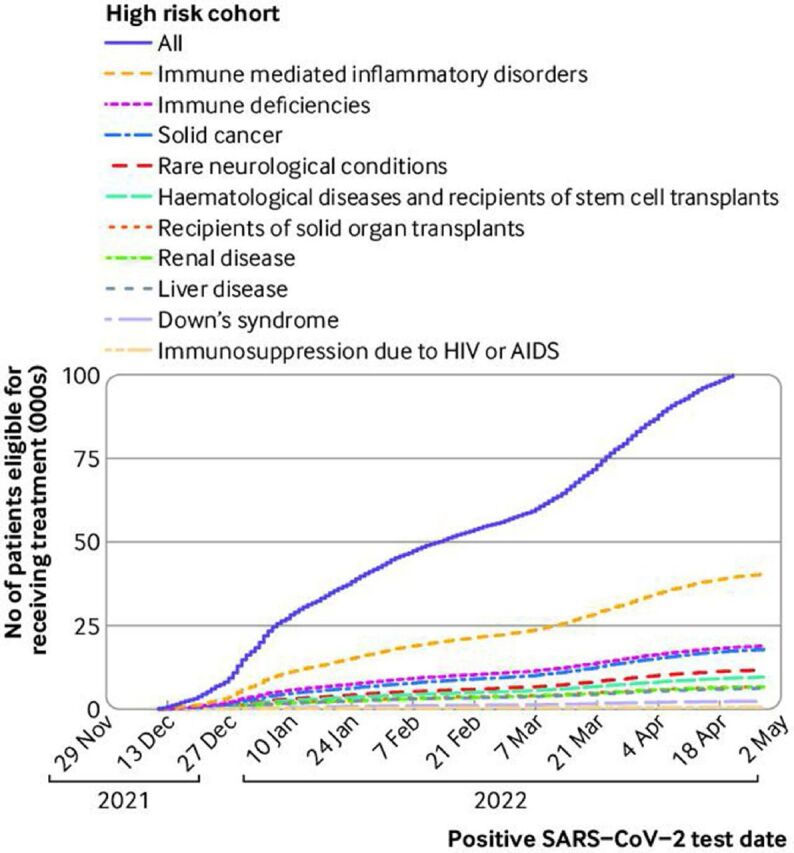
Cumulative total number of potentially eligible patients receiving antiviral drugs or neutralising monoclonal antibodies for covid-19 treatment since 11 December 2021, stratified by high risk group. Patients were considered eligible on the date of their positive SARS-CoV-2 test. Patients could appear in more than one high risk group, and the overall number in each group is likely to be an overestimation owing to the inclusion of SARS-CoV-2 infection confirmed by either lateral flow or polymerase chain reaction (PCR) test (where only infections confirmed by PCR should have been treated, according to guidance in effect before 10 February 2022), and potentially including patients with no symptoms

Between 11 December 2021 and 28 April 2022, 93 870 patients registered at a TPP practice in England were identified as potentially being eligible for receiving an antiviral or nMAB treatment for covid-19 (5010/93 870 patients were included because they had a record for receiving treatment, but who were not otherwise considered eligible). The number of patients potentially eligible in each high risk group is described in figure 1 and table 2, with the most potentially eligible patients classified as having an immune mediated inflammatory disorder (n=37 140).

**Table 2:** Number and proportion of potentially eligible patients in OpenSAFELY-TPP who received treatment for covid-19 between 16 December 2021 and 28 April 2022, by high risk group and treatment type

High risk group	Eligible (No)	Covid-19 treatment											
		All		Paxlovid		Sotrovimab		Remdesivir		Molnupiravir		Casirivimab/imdevimab	
		No	% (95% CI)	No	% (95% CI)	No	% (95% CI)	No	% (95% CI)	No	% (95% CI)	No	% (95% CI)
All	93 870	19 040	20 (20 to 21)	4680	25 (24 to 25)	9660	51 (50 to 51)	30	0 (0 to 0)	4620	24 (24 to 25)	50	0 (0 to 0)
Immune mediated inflammatory disorders	37 140	7910	21 (21 to 22)	1300	16 (16 to 17)	4400	56 (55 to 57)	10	0 (0 to 0)	2170	27 (26 to 28)	20	0 (0 to 0)
Immune deficiencies*	18 060	3230	18 (17 to 18)	810	25 (24 to 27)	1560	48 (47 to 50)	<8	—	850	26 (25 to 28)	10	0 (0 to 1)
Solid cancer	15 700	2810	18 (17 to 18)	400	14 (13 to 16)	1640	58 (57 to 60)	<8	—	770	27 (26 to 29)	10	0 (0 to 1)
Rare neurological conditions	11 090	2740	25 (24 to 26)	860	31 (30 to 33)	1230	45 (43 to 47)	<8	—	640	23 (22 to 25)	<8	—
Haematological diseases and recipients of stem cell transplants	8430	2760	33 (32 to 34)	460	17 (15 to 18)	1600	58 (56 to 60)	<8	—	680	25 (23 to 26)	10	0 (0 to 1)
Recepients of solid organ transplants	6260	2070	33 (32 to 34)	60	3 (2 to 4)	1480	71 (70 to 73)	<8	—	510	25 (23 to 26)	<8	—
Renal disease	5860	2020	34 (33 to 36)	<8	—	1460	72 (70 to 74)	<8	—	540	27 (25 to 29)	<8	—
Liver disease	5110	800	16 (15 to 17)	40	5 (3 to 7)	510	64 (60 to 67)	<8	—	250	31 (28 to 34)	<8	—
Down’s syndrome	2310	460	20 (18 to 22)	140	30 (26 to 35)	160	35 (30 to 39)	<8	—	160	35 (30 to 39)	<8	—
High risk group unknown	1330	1330	100 (100 to 100)	1280	96 (95 to 97)	50	4 (3 to 5)	<8	—	<8	—	<8	—
Immunosuppression due to HIV or AIDS	570	340	60 (56 to 64)	10	3 (1 to 5)	170	50 (45 to 55)	<8	—	160	47 (42 to 52)	<8	—

CI=confidence interval.Patients can appear in more than one risk group. Patient numbers of 0-7 are shown as <8, with remaining numbers rounded to the nearest 10; as a result percentages might not add up to 100%. High risk groups are arranged in descending order, according to number of potentially eligible patients.

*In the publication on which this analysis is based, this group was labelled “primary immune deficiency,”^10^ but in the present study this group has been labelled according to the current NHS Digital policy (version 5).

### Coverage of covid-19 treatment

**Figure 2:**
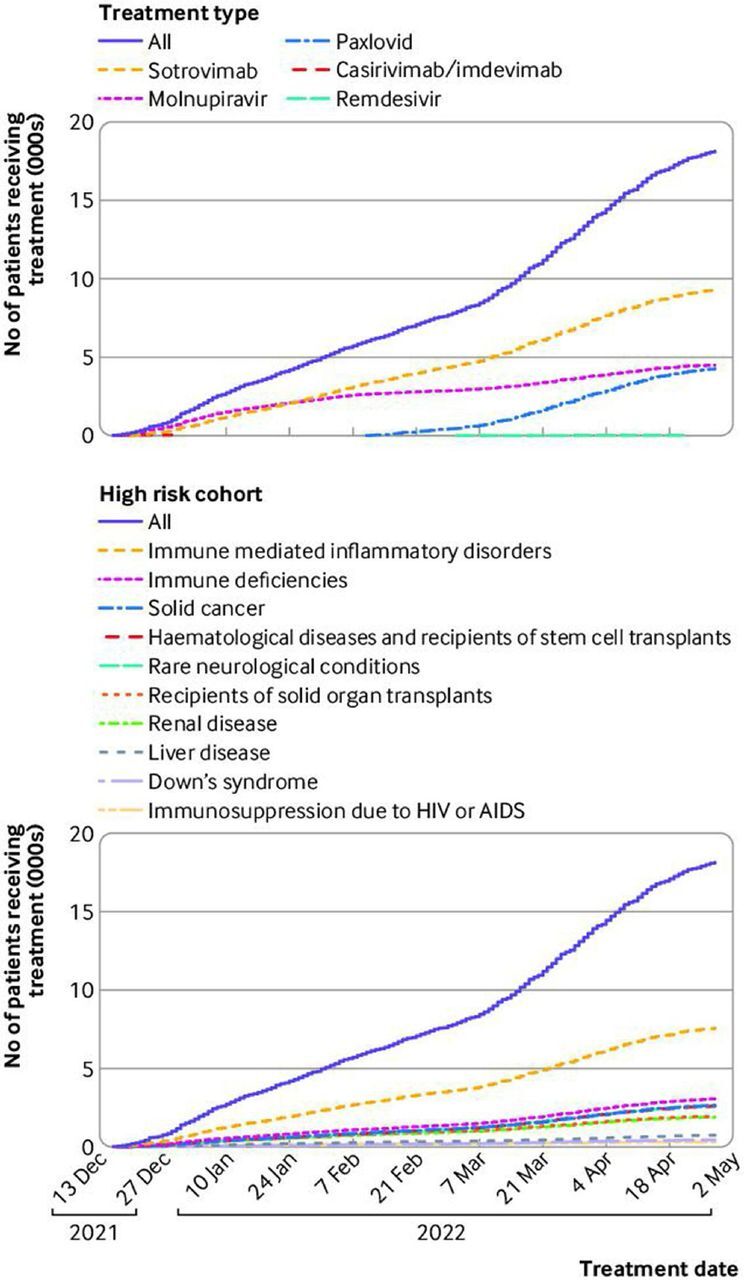
Cumulative total number of patients in OpenSAFELY-TPP who received antiviral drugs or neutralising monoclonal antibodies for covid-19 treatment since 16 December 2021, stratified by treatment type and high risk groups. Shorter lines for paxlovid and casirivimab/imdevimab reflect availability and guidance. A total of 330 treated patients were excluded because their date of treatment was after 28 April 2022; patients could appear in more than one high risk group

Of the 93 870 potentially eligible patients, 19 040 (20%) received treatment from a CMDU (table 2; figure 2; 560 patients were excluded owing to having records of multiple treatments within a two week period, or an implausible treatment date). The proportion of potentially eligible patients receiving treatment increased over time, from 9% (190/2220) in the first week of the roll-out to 29% (460/1600) in the last week of April 2022 (online supplemental figure S1). Sotrovimab was the most widely used treatment over the study period (n=9960, 51% of those treated) followed by paxlovid (n=4680, 25% of those treated) and molnupiravir (n=4620, 24% of those treated). Use of casirivimab/imdevimab (n=50) and remdesivir (n=30) was low. Of the 74 830 patients who were eligible but did not receive treatment, 2% (n=1500) were admitted to hospital within five days after their positive test, which could have been the reason for not being treated.

A sensitivity analysis restricting the population to only those patients with a symptomatic flag associated with their positive SARS-CoV-2 test reduced the potentially eligible population from 93 870 to 23 320 and the treated population from 19 040 to 4640. This decrease resulted in a 75% and 76% reduction, respectively—equivalent to 20% of patients receiving treatment from a CMDU.

### High risk patient groups

Of 661 609 people in a high risk group registered at a TPP practice, 93 870 were deemed potentially eligible for treatments and 96% (n=89 750) were assigned to at least one EHR derived high risk group, with an additional 3% (n=2790) assigned to at least one clinician assigned high risk group in the covid-19 therapeutics dataset. Of the 19 040 patients who received treatment, 78% (n=14 920) were assigned to at least one EHR derived high risk group, of whom almost all (98%, n=14 590) matched the clinician assigned high risk groups. Of the remaining 22% (n=4120) of patients who received treatment but had no EHR derived high risk group, the majority (82%, n=3390) had a clinician assigned high risk group of immune mediated inflammatory disorder, were primarily treated with paxlovid (for which the clinician assigned high risk group was not available), or had a clinician assigned high risk group of solid cancer (33% (n=1350), 31% (n=1280), and 18% (n=760), respectively).

The proportion of potentially eligible patients receiving treatment varied by high risk group (table 2). For example, 21% (95% confidence interval 21% to 22%) of patients in the largest eligible group (immune mediated inflammatory disorders) received treatment, and values ranged from 16% (15% to 17%) in liver disease to 60% (56% to 64%) in immunosuppression due to HIV or AIDS. The type of treatment given also varied by high risk group with sotrovimab favoured over molnupiravir or paxlovid in all but three high risk groups: Down's syndrome (35%; 30% to 39%), rare neurological conditions (45%; 43% to 47%), and immune deficiencies (48%; 47% to 50%).

### Key personal and clinical characteristics of treated patients

Table 3 shows the number and proportion of potentially eligible patients who received treatment for covid-19 by 28 April 2022, broken down by population and clinical categories and by treatment type. Among eligible patients who received treatment, patients were more likely to be white (21%; 95% confidence interval 21% to 21%) or Asian or Asian British (17%; 16% to 18%) than black or black British (11%; 10% to 12%). The percentage of patients treated correlated with deprivation, with 15% (14% to 15%) receiving treatment in the most deprived areas, compared with 23% (23% to 24%) in the least deprived areas; and similarly with rurality (16% (16% to 17%) for urban conurbation *v* 25% (24% to 25%) for rural village and dispersed). The East of England had the highest rate of treatment (25%; 24% to 25%), whereas Yorkshire and the Humber had the lowest rate (13%; 12% to 14%). Some clinical groups were much less likely to be treated (dementia 6% (5% to 7%), care home residents 6% (6% to 7%)), and others had a slightly reduced chance to be treated (patients with sickle cell disease 10% (8% to 12%), or those who had a severe mental illness 16% (14% to 18%)). Patients classed as clinically extremely vulnerable were slightly more likely to be treated (26%; 25% to 26%). Unvaccinated patients who had a SNOMED code associated with refusing vaccination were substantially less likely to receive treatment (7%; 6% to 9%).

**Table 3:** Number and proportion (%) of potentially eligible patients in OpenSAFELY-TPP who received treatment for covid-19 between 11 December 2021 and 28 April 2022, by population and clinical categories and by treatment type

Patient group and variable	No of potentially eligible patients	Treated											
		All		Paxlovid		Sotrovimab		Remdesivir		Molnupiravir		Casirivimab/imdevimab	
		No	% (95% CI)	No	% (95% CI)	No	% (95% CI)	No	% (95% CI)	No	% (95% CI)	No	% (95% CI)
All	93 870	19 040	20 (20 to 21)	4680	25 (24 to 25)	9660	51 (50 to 51)	30	0 (0 to 0)	4620	24 (24 to 25)	50	0 (0 to 0)
Age band (years)													
12-29	7610	1270	17 (16 to 18)	310	24 (22 to 27)	630	50 (47 to 52)	<8	—	320	25 (23 to 28)	10	1 (0 to 1)
30-39	10 640	2320	22 (21 to 23)	630	27 (25 to 29)	1150	50 (48 to 52)	<8	—	530	23 (21 to 25)	10	0 (0 to 1)
40-49	14 420	3280	23 (22 to 23)	870	27 (25 to 28)	1650	50 (49 to 52)	<8	—	740	23 (21 to 24)	10	0 (0 to 0)
50-59	18 900	4260	23 (22 to 23)	1120	26 (25 to 28)	2150	50 (49 to 52)	<8	—	980	23 (22 to 24)	10	0 (0 to 0)
60-69	17 760	3740	21 (20 to 22)	900	24 (23 to 25)	1970	53 (51 to 54)	<8	—	860	23 (22 to 24)	10	0 (0 to 0)
70-79	15 810	3040	19 (19 to 20)	670	22 (21 to 24)	1580	52 (50 to 54)	<8	—	780	26 (24 to 27)	<8	—
≥80	8720	1130	13 (12 to 14)	180	16 (14 to 18)	520	46 (43 to 49)	<8	—	420	37 (34 to 40)	<8	—
Sex													
Female	53 680	11 360	21 (21 to 22)	2990	26 (26 to 27)	5690	50 (49 to 51)	20	0 (0 to 0)	2640	23 (22 to 24)	20	0 (0 to 0)
Male	40 190	7680	19 (19 to 19)	1690	22 (21 to 23)	3970	52 (51 to 53)	10	0 (0 to 0)	1980	26 (25 to 27)	30	0 (0 to 1)
Unknown	<8	<8	—	<8	—	<8	—	<8	—	<8	—	<8	—
Ethnic group													
White	82 770	17 450	21 (21 to 21)	4330	25 (24 to 25)	8870	51 (50 to 52)	20	0 (0 to 0)	4180	24 (23 to 25)	40	0 (0 to 0)
Asian or Asian British	3840	660	17 (16 to 18)	130	20 (17 to 23)	370	56 (52 to 60)	<8	—	160	24 (21 to 28)	<8	—
Black or Black British	2910	320	11 (10 to 12)	70	22 (17 to 26)	140	44 (38 to 49)	<8	—	100	31 (26 to 36)	<8	—
Mixed	1180	180	15 (13 to 17)	30	17 (11 to 22)	90	50 (43 to 57)	<8	—	60	33 (26 to 40)	<8	—
Other ethnic groups	1030	190	18 (16 to 21)	40	21 (15 to 27)	100	53 (46 to 60)	<8	—	60	32 (25 to 38)	<8	—
Unknown	2150	220	10 (9 to 12)	80	36 (30 to 43)	80	36 (30 to 43)	<8	—	60	27 (21 to 33)	<8	—
Index of multiple deprivation group (divided by quintiles)													
1 (most deprived)	15 710	2290	15 (14 to 15)	460	20 (18 to 22)	1250	55 (53 to 57)	<8	—	570	25 (23 to 27)	<8	—
2	17 520	3220	18 (18 to 19)	710	22 (21 to 23)	1650	51 (50 to 53)	<8	—	850	26 (25 to 28)	10	0 (0 to 1)
3	19 960	4350	22 (21 to 22)	1000	23 (22 to 24)	2240	51 (50 to 53)	<8	—	1090	25 (24 to 26)	10	0 (0 to 0)
4	19 390	4260	22 (21 to 23)	1150	27 (26 to 28)	2090	49 (48 to 51)	<8	—	1010	24 (22 to 25)	10	0 (0 to 0)
5 (least deprived)	18 730	4390	23 (23 to 24)	1220	28 (26 to 29)	2170	49 (48 to 51)	10	0 (0–0)	990	23 (21 to 24)	10	0 (0 to 0)
Unknown	2560	520	20 (19 to 22)	140	27 (23 to 31)	260	50 (46 to 54)	<8	—	120	23 (19 to 27)	<8	—
Rurality													
Urban—conurbation	21 280	3450	16 (16 to 17)	690	20 (19 to 21)	2010	58 (57 to 60)	<8	—	740	21 (20 to 23)	<8	—
Urban—city and town	48 840	10 040	21 (20 to 21)	2540	25 (24 to 26)	4800	48 (47 to 49)	10	0 (0 to 0)	2650	26 (26 to 27)	30	0 (0 to 0)
Rural—town and fringe	12 460	2860	23 (22 to 24)	740	26 (24 to 27)	1470	51 (50 to 53)	<8	—	640	22 (21 to 24)	<8	—
Rural—village and dispersed	8830	2170	25 (24–to 25)	570	26 (24 to 28)	1120	52 (50 to 54)	<8	—	480	22 (20 to 24)	<8	—
Unknown	2470	510	21 (19 to 22)	130	25 (22 to 29)	260	51 (47 to 55)	<8	—	120	24 (20 to 27)	<8	—
Region													
East Midlands	16 150	3560	22 (21 to 23)	1070	30 (29 to 32)	2080	58 (57 to 60)	<8	—	390	11 (10 to 12)	20	1 (0 to 1)
East of England	23 160	5760	25 (24 to 25)	1050	18 (17 to 19)	2970	52 (50 to 53)	10	0 (0 to 0)	1700	30 (28 to 31)	20	0 (0 to 0)
London	4880	890	18 (17 to 19)	190	21 (19 to 24)	390	44 (41 to 47)	<8	—	310	35 (32 to 38)	<8	—
North East	4390	710	16 (15 to 17)	120	17 (14 to 20)	490	69 (66 to 72)	<8	—	100	14 (12 to 17)	<8	—
North West	8940	1720	19 (18 to 20)	520	30 (28 to 32)	870	51 (48 to 53)	<8	—	340	20 (18 to 22)	<8	—
South East	6820	1370	20 (19 to 21)	450	33 (30 to 35)	600	44 (41 to 46)	<8	—	330	24 (22 to 26)	<8	—
South West	13 850	2790	20 (19 to 21)	590	21 (20 to 23)	1270	46 (44 to 47)	<8	—	920	33 (31 to 35)	<8	—
West Midlands	3180	590	19 (17 to 20)	70	12 (9 to 14)	450	76 (73 to 80)	<8	—	70	12 (9 to 14)	<8	—
Yorkshire and the Humber	12 320	1600	13 (12 to 14)	600	38 (35 to 40)	540	34 (31 to 36)	<8	—	460	29 (27 to 31)	<8	—
Unknown	180	40	22 (16 to 28)	20	50 (35 to 65)	20	50 (35 to 65)	<8	—	<8	—	<8	—
Additional clinical risk groups													
Autism	560	130	23 (20 to 27)	30	23 (16 to 30)	60	46 (38 to 55)	<8	—	40	31 (23 to 39)	<8	—
Care home resident	3400	220	6 (6 to 7)	50	23 (17 to 28)	40	18 (13 to 23)	<8	—	130	59 (53 to 66)	<8	—
Dementia	2180	140	6 (5 to 7)	20	14 (8 to 20)	50	36 (28 to 44)	<8	—	70	50 (42 to 58)	<8	—
Learning disability	2750	540	20 (18 to 21)	160	30 (26 to 33)	190	35 (31 to 39)	<8	—	180	33 (29 to 37)	<8	—
Serious mental illness	1170	190	16 (14 to 18)	30	16 (11 to 21)	100	53 (46 to 60)	<8	—	60	32 (25 to 38)	<8	—
Housebound	2420	480	20 (18 to 21)	100	21 (17 to 24)	210	44 (39 to 48)	<8	—	160	33 (29 to 38)	<8	—
Clinically extremely vulnerable	49 580	12 790	26 (25 to 26)	2730	21 (21 to 22)	6860	54 (53 to 54)	20	0 (0–0)	3160	25 (24 to 25)	40	0 (0 to 0)
Sickle cell disease	990	100	10 (8 to 12)	40	40 (30 to 50)	30	30 (21 to 39)	<8	—	30	30 (21 to 39)	<8	—
Vaccination status													
Unvaccinated (refused)	1090	80	7 (6 to 9)	20	25 (16 to 34)	40	50 (39 to 61)	<8	—	20	25 (16 to 34)	<8	—
Unvaccinated	3190	260	8 (7 to 9)	50	19 (14 to 24)	120	46 (40 to 52)	<8	—	80	31 (25 to 36)	<8	—
One vaccination	2120	270	13 (11 to 14)	70	26 (21 to 31)	130	48 (42 to 54)	<8	—	60	22 (17 to 27)	<8	—
Two vaccinations	10 000	1090	11 (10 to 12)	200	18 (16 to 21)	550	50 (47 to 53)	<8	—	330	30 (28 to 33)	10	1 (0 to 1)
Three or more vaccinations	77 470	17 350	22 (22 to 23)	4340	25 (24 to 26)	8820	51 (50 to 52)	20	0 (0 to 0)	4130	24 (23 to 24)	40	0 (0 to 0)

Patient numbers of 0-7 are shown as <8, with remaining numbers rounded to the nearest 10; as a result percentages might not add up to 100%.

### Consistency with guidance

Of 19 040 patients who received treatment for covid-19, 6% (n=1150) did not have evidence of a positive SARS-CoV-2 test, 22% (n=4120) did not have an EHR derived high risk group, and 1% (n=210) were admitted to the hospital on or before their date of positive test but were not discharged on or before that date. We found a small number of other potential inconsistencies with guidance for patients who received treatment (online supplemental figure S2), such as a potential contraindication—of the contraindications included, the only one identified in results was recorded adolescent weight ≤40 kg for sotrovimab (<1%; n=20) (see the discussion below).

Overall, of patients who received treatment, 95% (n=18 100) did so within the respective treatment-specific eligibility window as estimated from their positive SARS-CoV-2 test date (as symptom onset date was not consistently available) (online supplemental figures S3 and S4). Treatment occurred most commonly two days (31%; n=5820) after a patient’s positive SARS-CoV-2 test. We found minor variation between the three most common treatments: treatment with paxlovid occurred slightly earlier than molnupiravir and sotrovimab, with 68% (n=3160) versus 51% (n=2360) and 53% (n=5120) of patients being treated within two days of their positive test, respectively.

### Covid medicine delivery units

Sustainability and transformation plans (used as a proxy for CMDU) was available for 93 700 eligible patients and 19 000 treated patients, with a total of 31 plans identified and included (six were excluded owing to having <10% population coverage in TPP practices; 2370 eligible patients and 310 treated patients). The overall proportion of potentially eligible patients receiving treatment varied by sustainability and transformation plan (online supplemental figure S5), ranging from 15% at the first decile (ie, 10% of plans had a coverage of ≤15%) to 28% at the ninth decile (ie, 90% of plans had a coverage of ≤28%). In addition, the maximum weekly proportion of patients treated over the study period ranged from 20% at the first decile to 43% at the ninth decile.

## Discussion

### Summary of findings

The NHS in England rapidly established CMDUs to support delivery of covid-19 treatments to people in the community from December 2021. In our study, of 93 870 patients likely to be eligible based on national clinical criteria, 19 040 (20%) received an antiviral drug or nMAB between 16 December 2021 and 28 April 2022. Sotrovimab was the most widely used treatment, followed by paxlovid and molnupiravir (51%, 25%, and 24%, respectively), although use varied over time. Use of other treatments was limited, reflecting availability and guidance. The proportion of the potentially eligible patients receiving treatment increased over time, rising from 9% in the first week of treatment availability to 29% in the latest week, and varied by high risk group (eg, 33% in recipients of solid organ transplants, 16% in liver disease). Treatment type also varied by high risk group, with sotrovimab favoured over molnupiravir and paxlovid in all but three high risk groups: Down's syndrome (35%), rare neurological conditions (45%), and immune deficiencies (48%). We observed differences in treatment rates between population and clinical subgroups. Those patients living in more socioeconomically deprived areas generally had lower treatment coverage (15% in the most deprived group *v* 23% in the least deprived group), as did those living in care homes (6%). Patients who were white or Asian or Asian British were most likely to receive treatment, with black or black British patients the least likely to receive treatment (21% and 17% *v* 11%, respectively). People aged 80 years and over had lower treatment coverage than those aged 50-59 years (13% *v* 23%). We observed substantial geographical variation in treatment rates between NHS regions (ranging from 13% in Yorkshire and the Humber to 25% in the East of England) and between sustainability and transformation plans (ranging from ≤15% in the first group to ≤28% in the top group).

### Strengths and weaknesses

Key strengths of this study were the scale, detail, and completeness of the underlying raw EHR data. The OpenSAFELY-TPP platform currently runs analyses across the full dataset of all raw, pseudonymised, single event level, clinical events for all 23.4 million patients. The General Practice Extraction Service dataset available in NHS Digital is a subset of these raw data created through a series of processing rules for specific aspects of general practice records applied at source before extraction. OpenSAFELY-TPP also provides data in near real time, providing unprecedented opportunities for audit and feedback to rapidly identify and resolve concerns around health service activity and clinical outcomes related to the covid-19 pandemic. The delay from entry of a clinical event into the EHR to its appearance in the OpenSAFELY-TPP platform varies from two to nine days, and is substantially faster than any other source of comprehensive general practice data.

We recognise some limitations to our analysis. Our population, although very large, is geographically clustered as a result of the geographical clustering in the EHR system used by general practices, and only 17% of general practices in London use TPP software. However, OpenSAFELY-TPP has been shown to be broadly representative of the English population.^24^ There are no a priori reasons to expect that this geographical clustering in our data will substantially affect estimates of the coverage of nMABs and antiviral drugs in England and its variation in key clinical and population groups.

As with other analyses of the same data, the newly sourced covid-19 therapeutics data represent treatment notifications, not prescriptions, and as such some data might have been missed because of delays in paperwork being completed. In addition, these data do not allow us to determine the reasons why some patients who were considered eligible might not have received treatment. Many patients who did not have symptoms or were improving clinically would not have been treated, which cannot be elucidated from the data.

Additionally, the codelists used are inclusive but not specific, and as a consequence these groups do not represent strict clinical groupings. People identified as potentially eligible in our study mightnot be in the identified at-risk group because of overinclusion within the NHS Digital codelists used (eg, immune deficiencies). A service evaluation of CMDUs in four regions across England^25^ showed that the most common reason for being ineligible on presentation to CMDUs was not being in an at-risk clinical group. The service evaluation found that 17% of the patients referred to CMDUs were judged eligible for treatment, which is in line with the coverage found in our study (20%).

EHR data might not always fully capture some eligibility criteria, and as such could underestimate the true number of eligible people in some groups or misclassify some people, particularly those identified through non-digital routes (eg, patients with kidney disease). And as previously described, we might not have ascertained all people in the group with immunosuppression due to HIV/AIDS because of specific arrangements around HIV data.^26^ In addition, our ascertainment of eligibility status might sometimes deviate from NHS Digital ascertained eligibility status on specific patients for two reasons. Firstly, OpenSAFELY has different and more detailed primary care records available; and secondly, when translating information from the NHS Digital website into analytical code, we had to make pragmatic decisions to resolve some discrepancies (as described in online supplemental table S1). As an example, 5010 of 19 040 people treated were not identified as eligible for treatment in our data, possibly because of false negatives due to missing data rather than substantial deviation from the guidance by CMDUs. Because of our decision to include people who were treated and erroneously classified as ineligible in our numerators but with the impossibility of including people who were untreated and erroneously classified as ineligible in our denominator, the estimated coverages of nMABs and antiviral drugs might be inflated. We have notified NHS Digital of discrepancies we identified with their codelists—which we regard as normal and expected with complex cohorting work—and additionally made all our analytical code and codelists openly available for inspection, as with all OpenSAFELY analyses.

Finally, our findings on apparent inconsistencies with treatment guidance should be taken as indicative only, because of several limitations. For instance, the date of treatment might occasionally be entered incorrectly on the submitted form; some SARS-CoV-2 test records might not pass into EHRs, and hospital admission related to covid-19 is difficult to determine accurately in Hospital Episode Statistics data (because it is not possible to fully determine whether the patient was treated for covid-19 while in hospital or if it was an incidental finding). Furthermore, treatment notification forms might not be promptly submitted, and the latest body weight recorded in EHR (required to be >40 kg for adolescents treated with sotrovimab or remdesivir) might not be current at the time of treatment.

### Policy implications and future research

To our knowledge, this paper is the first study to describe in detail the personal and clinical features of those who received treatments from CMDUs across England; and the first to report variation in treatment by detailed personal and clinical characteristics. Our finding that only 20% of potentially eligible patients received treatment could be of concern, although this proportion might only reflect the limitations discussed above. Although sotrovimab was the first line treatment available during the majority of the study period, almost 50% of patients received molnupiravir or paxlovid; this finding might be explained by sotrovimab requiring an infusion in a clinic setting, presenting greater logistical challenges. Focusing on the three groups (Down's syndrome, rare neurological conditions, and immune deficiencies) where sotrovimab was not first choice might be a pragmatic first step for investigations on the reasons for these choices.

Paxlovid was implemented as a first line treatment next to sotrovimab on 10 February 2022, reflected by a slowing in the uptake of molnupiravir from that date.^11^ Both the antiviral drugs molnupiravir and paxlovid are used orally.^13^ The low uptake of remdesivir might be explained by logistical challenges, because remdesivir is given intravenously in a three day course.^11^ In addition, concerns were raised about the low efficacy of sotrovimab against the omicron BA.2 sublineage,^27^ the dominant circulating variant from mid-February.^28^ Our findings of discrepancies in treatment between different groups is notable and consistent with our analysis of sociodemographic and ethnic group variation in the receipt of covid-19 vaccinations.^14^ Additionally, the finding of lower coverage among those patients aged ≥80 years requires investigation, particularly because age is associated with covid-19 death and admission to hospital.^29^

Further research and investigation is required to understand and deal with the causes of any inequity, such as the identification of barriers to the uptake of covid-19 vaccines.^30^ Additionally, understanding variation in the choice of individual treatment option beyond clinical indications and cautions will be of utmost importance as more information on the relative efficacy emerges from ongoing trials.

Finally, all our analytical code is openly available for reuse and can be used to underpin observational work on clinical effectiveness and safety of treatments. To our knowledge, no head-to-head comparative interventional research has been reported to date for these treatments. The effectiveness of these covid-19 treatments is supported by the original randomised controlled trials.^31–33^ Observational studies are starting to emerge on the effectiveness of these treatments in the clinical setting.^34–39^ The timeliness, granularity, and scale of the data available via OpenSAFELY offers unprecedented opportunity to carry out rapid observational work to inform the roll-out of new treatments.

The reasons underpinning variation in treatments delivered by CMDUs are not yet understood, and information presented here should not be misinterpreted as a criticism of the rapidly established CMDUs in the context of high levels of infection,^40^ but rather as an example of the value of rapid turnaround data monitoring to help optimise the successful delivery of an ambitious national treatment programme. We will produce routine data updates at https://reports.opensafely.org/ to assist with ongoing monitoring and targeted initiatives to resolve gaps in coverage as well as informing development of study designs on the efficacy and safety of these new treatments.

### Conclusions

The NHS in England has rapidly deployed facilities to offer novel therapeutics for the rapid treatment of covid-19 in the community. Targeted activity might be needed to resolve lower treatment rates observed among certain geographical areas and key groups including ethnic minority groups, people aged ≥80 years, people living in areas of higher deprivation, and care home residents. Near real time data monitoring can help support individuals on the front line making complex operational decisions around treatment delivery.

## Data Availability

Data may be obtained from a third party and are not publicly available. Access to the underlying identifiable and potentially re-identifiable pseudonymised electronic health record data is tightly governed by various legislative and regulatory frameworks, and restricted by best practice. The data in OpenSAFELY are drawn from general practice data across England where TPP is the data processor. TPP developers (CB, JC, and SH) initiate an automated process to create pseudonymised records in the core OpenSAFELY database, which are copies of key structured data tables in the identifiable records. These are linked onto key external data resources that have also been pseudonymised via SHA-512 one-way hashing of NHS numbers using a shared salt. Bennett Institute for Applied Data Science developers and principal investigators (BG, CEM, SCJB, AJW, WJH, HJC, DE, PI, SD, GH, RMS, ID, TW, JM, MW, RYP, KB and CTR) holding contracts with NHS England have access to the OpenSAFELY pseudonymised data tables as needed to develop the OpenSAFELY tools. These tools in turn enable researchers with OpenSAFELY Data Access Agreements to write and execute code for data management and data analysis without direct access to the underlying raw pseudonymised patient data, and to review the outputs of this code. All code for the full data management pipeline—from raw data to completed results for this analysis—and for the OpenSAFELY platform as a whole is available for review at github.com/OpenSAFELY.
